# Estimating the public health impact of disbanding a government alcohol monopoly: application of new methods to the case of Sweden

**DOI:** 10.1186/s12889-018-6312-x

**Published:** 2018-12-22

**Authors:** Tim Stockwell, Adam Sherk, Thor Norström, Colin Angus, Mats Ramstedt, Sven Andréasson, Tanya Chikritzhs, Johanna Gripenberg, Harold Holder, John Holmes, Pia Mäkelä

**Affiliations:** 1 Canadian Institute for Substance Use Research (CISUR), Department of Psychology, University of Victoria, PO Box 1700 STN CSC, Victoria, BC V8W 2Y2 Canada; 2 Canadian Institute for Substance Use Research (CISUR), Social Dimensions of Health Program, University of Victoria, Victoria, BC Canada; 3 Swedish Institute for Social Research, Stockholm University, Stockholm, Sweden; 4 University of Sheffield, Sheffield, UK; 5 The Swedish Council for Information on Alcohol and Other Drugs (CAN), Stockholm, Sweden; 6 Department of Public Health Sciences, Karolinska Institutet, Stockholm, Sweden; 7 Health Sciences, National Drug Research Institute, Curtin University, Perth, Australia; 8 Department of Clinical Neuroscience, Stockholm Prevents Alcohol and Drug Problems (STAD), Karolinska Institutet, Stockholm, Sweden; 9 Prevention Research Center, Pacific Institute for Research and Evaluation, Berkeley, CA USA; 10 University of Sheffield, Sheffield, UK; 11 National Institute for Health and Welfare, Helsinki, Finland

**Keywords:** Alcohol monopoly, Sweden, Mortality, Morbidity, Privatisation, Policy modelling

## Abstract

**Background:**

Government alcohol monopolies were created in North America and Scandinavia to limit health and social problems. The Swedish monopoly, Systembolaget, reports to a health ministry and controls the sale of all alcoholic beverages with > 3.5% alcohol/volume for off-premise consumption, within a public health mandate. Elsewhere, alcohol monopolies are being dismantled with evidence of increased consumption and harms. We describe innovative modelling techniques to estimate health outcomes in scenarios involving Systembolaget being replaced by 1) privately owned liquor stores, or 2) alcohol sales in grocery stores. The methods employed can be applied in other jurisdictions and for other policy changes.

**Methods:**

Impacts of the privatisation scenarios on pricing, outlet density, trading hours, advertising and marketing were estimated based on Swedish expert opinion and published evidence. Systematic reviews were conducted to estimate impacts on alcohol consumption in each scenario. Two methods were applied to estimate harm impacts: (i) alcohol attributable morbidity and mortality were estimated utilising the International Model of Alcohol Harms and Policies (InterMAHP); (ii) ARIMA methods to estimate the relationship between per capita alcohol consumption and specific types of alcohol-related mortality and crime.

**Results:**

Replacing government stores with private liquor stores (Scenario 1) led to a 20.0% (95% CI, 15.3–24.7) increase in per capita consumption. Replacement with grocery stores (Scenario 2) led to a 31.2% (25.1–37.3%) increase. With InterMAHP there were 763 or + 47% (35–59%) and 1234 or + 76% (60–92%) more deaths per year, for Scenarios 1 and 2 respectively. With ARIMA, there were 850 (334–1444) more deaths per year in Scenario 1 and 1418 more in Scenario 2 (543–2505). InterMAHP also estimated 10,859 or + 29% (22–34%) and 16,118 or + 42% (35–49%) additional hospital stays per year respectively.

**Conclusions:**

There would be substantial adverse consequences for public health and safety were Systembolaget to be privatised. We demonstrate a new combined approach for estimating the impact of alcohol policies on consumption and, using two alternative methods, alcohol-attributable harm. This approach could be readily adapted to other policies and settings. We note the limitation that some significant sources of uncertainty in the estimates of harm impacts were not modelled.

## Background

### Rationale for present study

Government monopolies for the sale or distribution of alcohol exist in North America (USA and Canada), Northern Europe and India. The North American alcohol monopolies were set up in the 1920’s and 1930’s, in most cases following the repeal of prohibition. Today, 17 US states control sales of spirits and/or wine at the wholesale level and 13 of these also at the retail level [1]. Retail “monopolies” for all alcohol beverages remain in twelve of Canada’s thirteen regional jurisdictions [2], though, increasingly, sales of alcohol are also being allowed in private stores and even grocery stores in some provinces [3]. In the Nordic countries, Iceland, Norway, Sweden and Finland have state alcohol retail monopolies for higher strength beers, wine and spirits. The state monopolies on import, distribution and wholesale distribution in these countries were abolished in Sweden and Finland in 1995 after entering EU, and in Norway in 1996 [4].

The Swedish government alcohol monopoly, Systembolaget, was established as a state owned national company in 1955 with a monopoly on the retail sale of alcoholic beverages in Sweden with a strength greater than 3.5%. Systembolaget has an explicit mandate to reduce alcohol-related harm, operates without a profit motive and reports to the Ministry of Health and Social Affairs. With increasing pressure to privatise or gradually dismantle government monopolies in other countries [5–7], it is important to use best available research evidence to estimate the likely impacts on public health and safety that would ensue under different privatisation scenarios. The purpose of the present study is to estimate the likely public health consequences were Systembolaget to be abolished, an issue last addressed in a 2008 study reported both by Norström et al. [8] and Holder et al. [9]. We present an innovative approach to estimating the changes in alcohol consumption and the associated public health burden that would result from two alternative policy scenarios in Sweden.

#### Swedish alcohol policy context

The legal age limit for selling alcoholic beverages off-premise (at Systembolaget) is 20 years and is 18 years for on-premise sales (restaurants, bars, cafes). Systembolaget currently runs 436 retail stores and licenses about 500 agents in rural areas to handle local distribution of alcohol. Rural agent stores account for less than 1 % of all sales. Systembolaget stores mostly open for 9 h on weekdays, for five hours on Saturdays and are closed on Sundays. Retail prices are based on the wholesale purchase price plus a basic fixed surcharge and a 19% surcharge on purchase price before alcohol taxes. Prices are fixed to be the same in all stores. Beer up to 3.5% alcohol by volume can also be sold in ordinary grocery stores, convenience stores and gasoline stations. Alcohol purchases over the Internet from foreign sellers, some with Swedish stakeholders, have been made legal but sales from this source remain below 1% of total sales [10].

In the Swedish parliament, all parties, with varying degrees of enthusiasm, support restrictive alcohol policies in order to limit alcohol consumption and harm. These policies include high alcohol taxes, a state owned retail monopoly, high age limits for alcohol purchase, restricting the number of licensed premises for alcohol serving and restricting marketing for alcohol. High alcohol taxes and the alcohol retail monopoly have received increased popular support in the last decade [11]. A number of other Swedish monopolies have been dismantled during the past decades, such as the railways, pharmacies and vehicle inspections. The gambling monopoly is also likely to be abolished soon. The alcohol retail monopoly thus has increasingly become the exception to the rule.

### Swedish alcohol consumption and related harm

Alcohol consumption has declined in Sweden from a peak in consumption in 2004 of 10.5 l per person above 15 years of age, to 9.2 l in 2016 based on official alcohol sales data [10]. Drinking among young people has gone down, while consumption among older people, above 65 years, has increased. The National Board of Health and Welfare estimate that alcohol-related mortality increased between 1990 to 2005 but has since decreased. Over this period estimated hospital stays for alcohol-related illness have increased slightly but steadily.

### Previous studies of privatisation of alcohol retail monopolies

A group under the auspices of the US Centers for Disease Control conducted a systematic review of alcohol retail privatisation events up to December 2010 [12]. Following criteria for design suitability and validity, 17 studies of 12 privatisation events were selected for the review. The median increase in per capita sales of privatised beverages was 44.4% over all studies, ranging from 0 to 305%. More recently, studies of the partial privatization of alcohol in British Columbia, Canada over a period of a few years, indicated that an increasing proportion of liquor stores in private ownership assessed across 89 regions was associated with increased alcohol consumption [13, 14], alcohol attributable mortality [3] and morbidity [15]. In the latter study, the relationship held after controlling for changes in alcohol pricing policies.

### Opportunity created by new methods for estimating alcohol attributable harm

Recent developments for estimating alcohol attributable harm in the Global Burden of Disease studies include new methods to estimate the continuous prevalence distribution of alcohol consumption at different levels throughout a population [16]. These involve the combined use of both population surveys (which frequently underestimate total alcohol consumption) and estimates of per capita alcohol consumption based on official sales or taxation data. Further, it has been demonstrated that if one knows the proportion of drinkers in a population and can estimate overall per capita consumption, it is possible to reliably estimate the distribution of that consumption across the whole population e.g. proportions of light, moderate or heavy drinkers [16]. Specifically, it has been shown that the within country distribution of alcohol consumption assessed by self-report survey for more than 60 countries can be best described by a gamma distribution.

With the technical advances described below, it is now possible to estimate changes in alcohol attributable harm for a given change in the total consumption of alcohol. Such an approach was applied after estimating changes in the per capita consumption of alcohol in the Swedish population under different policy scenarios using the International Model of Alcohol Harms and Policies (InterMAHP) [17, 18], a new, open access resource to support the estimation of alcohol attributable harm. InterMAHP is based on similar principles to those used in Global Burden of Disease (GBD) estimates for alcohol and was created in collaboration with authors of the GBD alcohol methods. However, it was designed to provide a more accessible tool and methods for alcohol harm estimation and, as well, to enable estimation of the effects of changes in alcohol consumption on rates of alcohol attributable harm. In addition, we estimated changes in alcohol attributable mortality and crime using an alternative ARIMA method based on observed relationships over many decades between per capita consumption and these outcomes following methods used in earlier studies [8].

## Methods

The study team identified key policy levers that would potentially change under the two privatisation scenarios from comprehensive and systematic literature reviews on alcohol policy [12, 19] and past evaluations of Systembolaget [8]: hours and days of trading; average alcohol prices; minimum available alcohol prices; alcohol advertising and promotions; and provision of alcohol to young people. We also considered potential changes in cross-border purchases of alcohol. The two selected scenarios themselves represent major alternative privatised systems: (i) a more restrictive one in which alcohol is permitted to be sold only in privately owned liquor stores and (ii) a more liberal system in which alcohol can be sold in any grocery store. Estimation of impacts on public health and safety proceeded through the steps explained below.

### Step 1: The extent to which policy levers would change under privatisation scenarios

We employed comparisons with privatisation experiences in Scandinavia and North America informed by expert Swedish opinion to estimate the extent to which outlet density, days and hours of trading, average and minimum available prices of alcohol and promotions of all kinds would change under each of the 2 scenarios (see Table 1 for summary). While there is also evidence for private liquor stores being less strict in their checking of customer age-IDs and level of intoxication than are government-owned stores [20], we were unable to find an empirical basis upon which to estimate the effects on population consumption and therefore, conservatively, excluded these from the analysis. The studies used to inform these estimates were drawn from the systematic reviews identified below in Step 2 as well as the team’s knowledge of research in alcohol monopoly countries. In particular, we drew heavily on a systematic review of the impacts of privatisation events on alcohol sales to identify relevant studies [12] and recent studies of the impacts of opening increasing numbers of private liquor stores alongside government stores in the Canadian province of British Columbia [13, 14]. The existence of the two kinds of stores operating alongside each other is almost unique and allows direct comparison on issues such as pricing and trading hours.

**Table 1:** The estimated changes in key policy levers in two privatisation scenarios

Policy Lever	Scenario 1 – Private Liquor Stores	Scenario 2 – Grocery Stores
Population density of liquor stores	200% increase	1500% increase
Sunday trading	An extra 12 h day added	An extra 14 h day added
Extended hours	An increase of 44%	An increase of 68%
Mean prices	Beer + 4.9%	Beer + 2.4%
	Wine + 6.0%	Wine + 3.0%
	Spirits + 1.4%	Spirits + 0.7%
Minimum prices	Beer −19.9%	Beer −24.9%
	Wine −12.5%	Wine −15.6%
	Spirits −20.6%	Spirits −25.7%
Promotions	Half the inverse effect of a ban	Inverse of effect of a ban

#### Population density of liquor stores

In Scenario 1 we estimated a 3-fold increase in liquor stores based on Sweden’s recentexperience with privatising pharmacies and also Canadian experiences of privatisations [13, 14]. This equates to an additional 10 outlets per 100,000 population. Under Scenario 2 it was assumed that all of Sweden’s 6900 grocery stores would sell alcohol, equating to an additional 75 outlets per 100,000 population.

#### Days of sale

We assumed the addition of Sunday sales in both scenarios with 12 h for Scenario 1 and 14 h for Scenario 2.

#### Additional operating hours

We assumed specialty stores would open 12 h per day in Scenario 1 (for 72 versus 50 h, Monday to Saturday = + 44%) based on opening hours of private stores in British Columbia, Canada and 14 h per day in Scenario 2 based on trading hours of Swedish grocery stores (for 84 versus 50 h, Monday to Saturday = + 68%).

#### Alcohol prices

We assumed small *increases* in average prices based on the privatisation of alcohol in Alberta, Canada (4.9% beer, 6% wine, 1.4% spirits) [13], the clearest case of a complete privatisation event with estimated impacts on per capita alcohol consumption identified from our systematic review. For Scenario 1, we also estimated that this increase would be counter-acted by larger decreases in the minimum prices based on a survey of private versus government liquor store prices at which alcohol was available in British Columbia, Canada [21] (− 19.9% beer, − 12.5% wine, − 20.6% spirits). The only published empirical studies of the impacts of minimum pricing on consumption come from Canada. The authors had access to this price survey of private liquor stores that operate almost uniquely alongside government-controlled liquor stores permitting price comparisons for cheapest alcohol brands between the two sources. For Scenario 2, we drew on data from Washington, USA [22] reporting how price changes compared in private liquor stores versus grocery stores following a recent privatisation event. Based on that study, we estimated that the increase in mean grocery store prices in Scenario 2 would be half that in Scenario 1, but that minimum prices would be 25% lower in Scenario 2.

#### Promotions, advertising and marketing

While comprehensive and systematic reviews consistently identify promotions, advertising and marketing as important drivers of alcohol consumption [23], especially among youth, we were unable to identify a method to quantify the intensity of these activities. Instead, we elected to use an approach used by Norstrom [8] of extrapolating estimated impacts of an advertising ban on alcohol consumption. At the present time, there are considerable restrictions on advertising, marketing and promotions of alcohol in Sweden, and the monopoly operates without a profit motive. We estimated an effect size opposite to that observed for a complete ban on alcohol advertising in a study of US states [24] for Scenario 2 and 50% of that for Scenario 1.

### Step 2: The independent effect of each policy lever on recorded per capita alcohol consumption

Comprehensive systematic reviews and, where possible, meta-analyses, were completed to estimate the effect on per capita alcohol consumption of the above changes in: (1) alcohol outlet density, (2) days and hours of alcohol sale, (3) price and (4) advertising and are reported in full elsewhere while being briefly summarised here [25]. Quality criteria were applied to select studies with controlled before and after intervention analyses.

#### Density of liquor outlets

Of 754 relevant articles identified, only four met the quality inclusion criteria, three of which were population-level studies [14, 26, 27], the other individual-level [28]. Different measures of outlet density ruled out a meta-analysis. The scale of changes in density estimated to occur under the two scenarios (200 and 1500% respectively) were significantly larger than those reported in two of the identified studies. We reanalysed data from the other identified study [15] and found evidence that the effects of increasing outlet density on alcohol consumption obeyed a decay function such that smaller proportional effects were seen at higher levels of outlet density that were equivalent to what was predicted for Systembolaget. This finding was used to estimate consumption impacts of the different increases in outlet density for the two scenarios.

#### Days and hours of sale

Of 1514 relevant papers identified, only 7 met the quality inclusion criteria and were used to formulate the scenarios, six of which studied days of sale [29–34] and one of which studied hours of sale [35]. Across-study results were consistent and a meta-analysis indicated that an additional day of sale was associated with a 3.4% increase in total consumption. Estimates were also made for the effect on per capita consumption of the additional hours of trading each day from Monday to Saturday (22 h in Scenario 1, 34 h in Scenario 2). These were based on the effect size estimated for the effect of the addition of a whole extra day of trading assuming, in the absence of other evidence, a decay function in effect size similar to that for outlet density.

#### Prices

We took estimates of the price elasticity of demand for each beverage type (beer, wine and spirits) of − 0.79, − 0.57 and − 0.96 respectively from a Swedish study [36] and used these to calculate the impact of the change in mean price on consumption. As no Swedish minimum price elasticities exist, we applied beverage-specific price elasticities for changes in the minimum available price of alcohol, calculated from Saskatchewan, Canada [37] of − 1.387, − 0.511 and − 0.589 respectively.

#### Advertising, promotion and marketing

We assumed a direct effect on recorded consumption under each scenario, based on evidence from [24].

### Step 3: The collective impact of all policy levers on total per capita alcohol consumption

We combined these independent effect estimates for each policy lever assuming a simple additive effect each applied to the baseline estimate. Swedish data from 2001 to 2005 [38] was used to estimate substitution between recorded and unrecorded consumption, resulting in an estimated elasticity of unrecorded demand of − 0.197. This figure was combined with the estimated net change in recorded consumption [10].

### Step 4: Estimating the uncertainty around modelled changes in per capita consumption

To estimate uncertainty around each parameter, we collected standard errors or confidence intervals around the selected empirical estimates quantifying the relationships between each policy parameter (i.e. outlet density, days and hours of sale et cetera) and age 15+ per capita alcohol consumption. We used a Probabilistic Sensitivity Analysis (PSA) framework to take 10,000 random draws from the probability distribution around each parameter and combine obtained values to estimate overall effects on per capita consumption, as well as for 95% confidence intervals around the estimates of the change in mean consumptionfor each scenario. Normal distributions were assumed for each parameter and the analysis was conducted using Excel, version 16.

### Step 5: Impacts on alcohol-related harms under each scenario

Two alternative analytic approaches were applied to the estimation of the impacts of changes in per capita consumption of alcohol attributable harms. The first applies assumptions derived from the international epidemiological literature regarding risk relationships between consumption and harm for many disease and injury outcomes. The second bases estimates on observed relationships over many years in Sweden between level of alcohol consumption and alcohol related harms. Each has strengths and weaknesses. The purpose was to investigate how sensitive the estimates would be to different analytic approaches.

#### Method a: InterMAHP alcohol attributable fractions

Using methodological principles based on Global Burden of Disease studies, e.g. [39], the International Model of Alcohol Harms and Policies [18] was used to estimate, Sweden-specific Sweden-specific alcohol-attributable deaths and hospital stays for an expanded list of conditions (see Appendix Table 6) were estimated for each of ten population subgroups, defined by gender and age (15–34,35-64,65+) using the internet-based resource InterMAHP [17]. For a comprehensive description of methods to calculate alcohol-attributable morbidity and mortality, including treatment of all methods choices used to run InterMAHP, see Sherk et al. [18]. We used the dose-response relationships from [40] to calculate Swedish AAFs for IHD morbidity and mortality. The InterMAHP default functions and values for all other conditions were used. The binge drinking level was defined in Sweden as 60 g/day for both men and women and the InterMAHP capped relative risk extrapolation method was used.

#### Method B: ARIMA modelling of Swedish consumption and harm data

The expected change in harm associated with each of the 2 scenarios was based on estimates of the recent historical relation between per capita alcohol consumption and harm summarised in Norstrom and Ramstedt [41]. We focused on a broad range of harm indicators in order to obtain a comprehensive assessment of the projected changes in population drinking. We included cirrhosis mortality as this is the classical indicator of harmful effects of chronic heavy consumption, as well as injury mortality, which is likely to be linked to episodic intoxication drinking. Suicide is an extreme self-destructive behaviour for which alcohol’s direct involvement in any case is often hard to ascertain but which, in general, is often influenced by drinking [42]. Assaults and drink driving represent two important indicators of harm from others’ drinking. Data sources, statistical methods and reported relationships between alcohol consumption and these outcomes are detailed elsewhere [43]. ICD-codes for the causes of death included are listed in Appendix Table 7. Data were analysed by applying the technique of seasonal ARIMA-modelling or SARIMA (seasonal autoregressive integrated moving average model) [44]. Error-correction modelling (ECM) was used to explore lagged effects in the relationship between population consumption and liver cirrhosis [45].

ICD-10 code data to 3 digits (e.g. C00) for 2014 for all modelled conditions were obtained from the Swedish Health and Welfare Database (accessed at http://www.socialstyrelsen.se/statistics/statisticaldatabase/causeofdeath) for deaths and the National Board of Health and Welfare for hospital stays.

##### Estimating the distribution of alcohol consumption

Swedish survey data were used to estimate: (i) the prevalence of lifetime abstainers (≤ 1 drink ever), (ii) the prevalence of former drinkers (< 1 drink past year), (iii) the prevalence of current drinkers (≥1 drink past year) (iv) the prevalence of binge drinkers (> 60 g ethanol/day at least monthly) and (v) average daily consumption within the subgroup. Data were obtained from the Swedish Council for Information on Alcohol and Other Drugs (CAN) (prevalence of lifetime abstainers and former drinkers), the National Prospective Study of Substance Use and Harm [46] (mean daily consumption by sub-group) and the CAN Monitor Survey (binge drinking) [11].

These data were combined with Swedish per capita consumption data to create subgroup-specific per capita consumption estimates following the methods described elsewhere [47]. The distribution of drinkers in each subgroup was calculated using a one-parameter definition of the Gamma distribution [47]. We assumed a maximum level of consumption of 250 g ethanol per day corresponding to the mean levels of consumption observed in street-involved groups of dependent drinkers observed in Canada [48].

##### Estimating relative risk curves for alcohol attributable conditions

Conditions for which alcohol consumption had a causal impact were identified via standardized methodology [49] (see Appendix Table 6 for summary). Relative risk curves for these conditions were obtained from Rehm et al. and are similar to those used in the WHO 2018 Global Status Report on Alcohol and Health. To calculate Alcohol Attributable Fractions (AAFs) for partially alcohol attributable conditions, we used the following general formula:

$$ AAF=\frac{P_f\left[{RR}_f-1\right]+{\int}_{0.03}^{250}P(x)\left[ RR(x)-1\right] dx}{P_f\left[{RR}_f-1\right]+{\int}_{0.03}^{250}P(x)\left[ RR(x)-1\right]\  dx+1} $$

where P_f_ is the prevalence of former drinkers, RR_f_is the relative risk of former drinkers, P(x) is the prevalence of drinkers at daily consumption level *x* and is calculating using the Gamma distribution, RR(x) is the disease-specific relative risk at daily consumption level *x* and 250 g is an assumed maximum daily consumption level [18].

##### Changes in the prevalence of “binge” drinking

Special AAFs were calculated for injuries, ischaemic stroke and ischaemic heart disease that took account of the prevalence of “binge drinking” as measured by survey data. Estimated changes from baseline in the prevalence of binge drinking due to increased consumption in each scenario were extrapolated from observed relationships between rates of binge drinking and mean daily consumption across the 10 age-gender sub-groups.

##### Wholly alcohol attributable conditions

Some conditions (e.g. mental and behavioural disorders due to alcohol, ICD10 code F10) are completely, and not partially, attributable to alcohol (i.e. its AAF = 1.00). For each population subgroup, an absolute risk function was calibrated, assuming a linear form, to match the observed number of deaths or hospital stays given the initial distribution of consumption. These functions were combined with the post-intervention distribution of consumption in order to estimate changes in the relevant harm outcomes under each scenario. See [50] for more details of the calibration process.

##### Changes in deaths and hospital stays

The percentage increases in per capita alcohol consumption were applied to consumption for each subgroup in both scenarios. We assumed the prevalence of abstainers and former drinkers would not change. Different distributions of current drinkers were then calculated using these updated per capita consumption figures for each scenario. These updated distributions of consumption were applied to the AAF formula above and updated AAFs were calculated for each condition, subgroup and scenario. An adjustment was also calculated to modify the number of hospital stays (or deaths) due to this increased consumption, calculated as

$$ {AAH}_1={H}_1\times {AAF}_1=\frac{H_0\left(1-{AAF}_0\right){AAF}_1}{1-{AAF}_1} $$

where H_1_ is the number of hospital stays for a condition under Scenario 1, H_0_ is number of hospital stays observed in 2014 (base case), and AAF_1_ and AAF_0_ are the AAFs calculated under Scenario 1 and the base case, respectively.

##### Statistical analysis

InterMAHP v1.0, an open access SAS-based software program, was used to perform the data analysis to calculate AAFs [17, 51] which were subsequently used to calculate the number of deaths and hospital stays that are attributable to alcohol consumption from the total number of recorded deaths and admissions for each condition.

## Results

### Effects of changes in policy levers on per capita alcohol consumption

The combined results of Steps 1 to 4 are shown in Table 2 with estimated effects of each individual policy change and their combined effects on per capita consumption with 95% confidence intervals.

**Table 2:** Estimated 95% Confidence Intervals around changes in recorded per capita consumption for each lever and overall change in consumption for each scenario

Lever	Scenario 1	Scenario 2
Density of outlets	9.47% *(7.44–11.58%)*	16.43% *(14.71–18.19%)*
Sunday trading	1.01% *(−3.21–5.27%)*	1.18% *(CI -3.70-6.24%)*
Extended opening hours	3.83% *(3.31–4.36%)*	4.82% *(CI 4.15–5.48%)*
Mean price	−2.83% *(− 3.91%- -1.73%)*	−1.41% *(− 1.96%- -0.88%)*
Minimum price	13.34% *(10.24–16.44%)*	16.67% *(12.86–20.55%)*
Promotions	2.50% *(0.27–4.75%)*	5.00% *(0.58–9.50%)*
Overall change in per capita consumption (recorded & unrecorded)	19.99% *(15.34–24.73%)*	31.23% *(25.12–37.33%)*

**Fig. 1:**
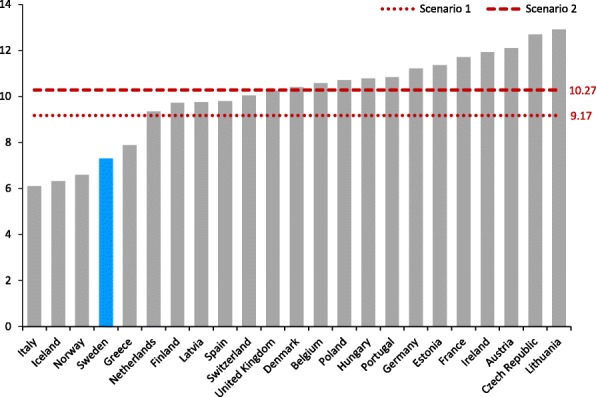
Per capita recorded alcohol consumption in 23 European countries (litres per year)

The estimates for revised levels of total per capita consumption are illustrated in Fig. 1 alongside consumption levels for 23 European countries in 2010, accessed from the European Commission public health indicators website (http://ec.europa.eu/health/alcohol/indicators_en). As can be seen, the estimates of consumption under both scenarios are well within the limits observed for other European countries with private alcohol retail systems, such as Denmark and Germany.

### Effects of changes in per capita alcohol consumption on levels of harms

#### Estimates from method a: InterMAHP attributable fractions

As shown in Table 3, the 20% increase in per capita consumption in Scenario 1 is predicted to lead to 763 additional AA deaths per year, an increase of 47% (95% CIs: 35–59%). The estimated 31.23% increase in per capita consumption in Scenario 2 is projected to cause an additional 1234 deaths per year, an increase of 76% (95% CIs, 60–92%). Alcohol may provide a protective effect for certain conditions such as hypertension, ischaemic heart disease, ischaemic stroke and type 2 diabetes, although this has been increasingly questioned [52]. This traditionally assumed protective effect, however, was taken into account and explains the negative number of AA deaths for cardiovascular conditions and diabetes.

**Table 3:** The estimated impacts of each privatisation scenario on alcohol-related harm based on the International Model of Alcohol Harms and Policies

Harm measure	Total Sweden 2014	Scenario 1 extra^a^ *(95% CIs)*	Scenario 2 extra^a^ *(95% CIs)*
*Alcohol attributable deaths*			
Cancers	712	138 *(106, 172)*	219 *(175, 263)*
Mental health	243	50 *(40, 59)*	70 *(59, 78)*
Cardiovascular	− 452	305 *(226, 391)*	516 *(398, 641)*
Digestive	394	134 *(100, 169)*	220 *(172, 270)*
Injuries	651	119 *(91, 145)*	183 *(147, 215)*
Infectious diseases	80	17 *(13, 22)*	27 *(22, 33)*
Type 2 diabetes	−133	−6 *(−5, −7)*	−9 *(−7, − 10)*
Total deaths: N *(95%*	1629	763 *(576–957)*	1234 *(974, 1501)*
*CIs)* % Change *(95% CIs)*	–	+ 47% *(35, 59%)*	+ 76% *(60, 92%)*
*Alcohol attributable hospital stays*			
Cancers	3068	668 *(509, 832)*	1060 *(846, 1277)*
Mental health	28,172	5635 *(4513, 6661)*	7874 *(6741, 8807)*
Cardiovascular	− 7934	1574 *(1193, 1970)*	2525 *(2002, 3053)*
Digestive	1972	550 *(415, 693)*	896 *(705, 1094)*
Injuries	10,565	1928 *(1478, 2361)*	2973 (*2398, 3507)*
Infectious diseases	2249	503 *(385, 623)*	790 *(633, 947)*
Type 2 diabetes	− 373	−12 *(−9, −14)*	−16 (*− 14, − 18)*
Total stays: N *(95% CIs)*	38,091	10,859 *(8493, 13,140)*	16,118 *(13,325, 18,685)*
% Change *(95% CIs)*	–	+ 29% *(22, 34%)*	+ 42% *(35, 49%)*

^a^Calculated as percentage change in alcohol attributable conditions

As shown also in Table 3, in Scenario 1 an additional 10,859 hospital stays per year was estimated, a 29% (95% CIs: 22–34%) increase. The 31% per capita consumption increase associated with Scenario 2 was projected to lead to 16,118 additional hospital stays due to alcohol per year, a 42% (95% CIs: 35–49%) increase. In both scenarios, the largest net increase is projected for mental health conditions followed by injuries, cardiovascular and digestive conditions.

Estimates of increased alcohol-related deaths and hospital stays under each scenario were also analysed by gender and three age groups and are reported in Appendix Tables 8 and 9.

#### Estimates based on method B: ARIMA modelling

Results of the ARIMA modelling to estimate the relationships between per capita consumption for five harm indicators are shown in Table 4, based on analyses reported by Norstrom and Ramstedt [41].

**Table 4:** Estimated effects of per capita alcohol consumption (litres of ethanol) on harm rates in Sweden, 1987 to 2015

	Elasticity estimate^a^	95% CIs
Cirrhosis	0.170	*0.124–0.215*
Suicide	0.122	*0.071–0.174*
Injuries	0.106	*0.018–0.194*
Assaults	0.102	*0.081–0.122*
Drink driving	0.157	*0.086–0.228*

^a^The proportional change in a harm indicator for a 1 l increase in per capita alcohol consumption

Applying the estimated elasticities in Table 4 to the estimated changes in per capita consumption for each scenario resulted in estimates of increased mortality and crime as shown in Table 5.

**Table 5:** Estimated impacts of each privatisation scenario on alcohol-related harm based on ARIMA analyses of Swedish time series data

Harm measure	Total Sweden 2014	Scenario 1	Scenario 2
		N, % *(95% CIs)*	N, % *(95% CIs)*
Alcoholic cirrhosis deaths	429	160 *(111–211)*	273 *(186–371)*
		+ 37.2% *(25.9–45.2%)*	+ 63.7% *(43.3–86.5%)*
Injury deaths	1833	399 *(61–797)*	660 *(96–1384)*
		+ 21.8% *(3.3–45.5%)*	+ 36.0% *(5.3–75.5%)*
Suicide deaths	1142	291 *(161–436)*	485 *(261–750)*
		+ 25.5%)	+ 42.4% *(22.9–65.6%)*
Total deaths	3404	850 *(334–1444)*	1418 *(543–2505)*
		+ 25.0% *(9.8–42.4%))*	+ 41.7% *(16.0–73.6%)*
Assault crimes	83,324	17,407 *(13,549-21,225)*	28,680 *(22,063-35,369)*
		+ 20.9% *(16.3–25.5)*	+ 34.4% *(26.5–42.4%)*
Drink-driving	13,769	4669 *(2388-7273)*	7940 *(3900-12,903)*
		+ 33.9% *(17.3–52.8%)*	+ 57.7% *(28.3%93.7%)*

## Discussion

This paper presents estimates of the public health and safety impacts of abolishing Systembolaget under two alternative scenarios. The baseline estimates for Sweden in 2014 (implied by the changes estimated above) indicate the extent of existing alcohol-related harm in Sweden with estimates for 2014 of 2081 deaths and 46,026 hospital stays being directly attributable to alcohol per year if the contested health benefits of alcohol use are discounted [52]. We demonstrate two methods of estimating increases in alcohol related harms based on estimated changes in per capita alcohol consumption under different policy scenarios. These indicate substantial increases in alcohol attributable deaths, crimes and hospital admissions were Sweden to privatise its liquor monopoly.

In Scenario 1, we assumed Systembolaget stores were replaced by privately-owned speciality liquor stores and that annual alcohol consumption would increase by 20.0% from 9.2 l to 11.1 l per capita as a result. Using the InterMAHP burden of disease approach, we estimated that Scenario 1 would lead to 763 additional deaths (+ 47%) and 10,859 additional hospital stays (+ 29%) per year. The ARIMA method provides alternative estimates for a narrower range of important alcohol-related harms. Using the ARIMA method, we estimate that each year under Scenario 1 there would be 160 (37.2%) more liver cirrhosis deaths, 399 (21.8%) more deaths from injuries, 291 (25.5%) more suicides, 17,407 (20.9%) more assaults and 4669 (33.9%) more drink driving offences.

In Scenario 2 (alcohol sold in privately-owned grocery stores), we estimate a 31.2% increase in alcohol consumption to an annual total of 12.2 l per capita adult. Using the InterMAHP methodology, this consumption increase would lead to 1234 more deaths each year (+ 76%) and 16,118 more hospital stays (+ 42%). Using the ARIMA method, we estimated there would be 273 (63.7%) more liver cirrhosis deaths, 660 (36.0%) more deaths from injuries, 485 (42.4%) more suicides, 28,680 (34.4%) more assaults and 7940 (57.7%) more drink driving offences.

### Differences from previous estimates

Norström et al. [8] estimated in the early 2000s the consequences of abolishing the Swedish alcohol monopoly. Despite the use of updated reviews of the published literature and analyses of recent Swedish data on alcohol-related harm, there are similarities with both the results and conclusions of the last published study by Norström et al. [8]. In both instances it was concluded that the density of liquor outlets, the hours that liquor stores are open, the average price of alcoholic products and the effects of marketing and promotion activities all have the potential to influence levels of alcohol consumption and related harms. Since the earlier report, there has been new research on floor or minimum prices. In the present exercise we estimate that while privatisation may slightly increase the average price of alcohol, this is more than offset by the effects on alcohol consumption of a reduction in the prices of the cheapest alcohol. In relation to impacts of all effects of privatisation on population consumption of alcohol, we estimated a larger impact for Scenario 1 (specialty liquor stores) than in Norström et al. [8] and a slightly smaller impact for Scenario 2 (grocery stores). The estimated changes in per capita alcohol consumption under each scenario are also well within the range reported in the main systematic review of privatisation events conducted by the US Centers for Disease Control [12], namely a median increase of 44.4% and range from 0 to 305%.

### Limitations and uncertainties

We acknowledge a range of factors that may have led us to overestimate, underestimate or have uncertain effects on our estimates. We assumed a simple additive effect such that the overall effect of the various policy changes is the sum of the individual effects as estimated from the published literature. There is only a small literature regarding how in practice the effects of policies are altered when they are introduced in combination. Studies from the US [53], Australia [54] and Canada [15] suggest that the combined effects of introducing two or more policies at the same time is a sub-additive effect on alcohol consumption i.e. the combined impact is less than the sum of the individual policy impacts. However, Norström et al. [8] argued that a multiplicative model is more applicable. In the absence of conclusive evidence, we took the middle course of assuming a simple additive model. Given the complexity and range of estimates reported in this paper and the absence of an empirical basis upon which to conduct a sub- additive model, we elected not to present sensitivity analyses here.

Our models follow the standard WHO GBD assumption that alcohol is protective in low doses for some cardiovascular conditions as well as type 2 diabetes. However, this assumption is being increasingly questioned for all-cause mortality [48], for cardiovascular disease [52] and type 2 diabetes [55] so we may have underestimated the net extent of alcohol-related harm in Sweden. There are also other general limitations to be acknowledged in relation to the widely used attributable fraction method. While Sweden specific attributable fractions were calculated based on systematic reviews of the international literature and meta-analyses describing risk relationships between alcohol consumption and diseases, it is possible that these risk relationships are different in Sweden. It should be noted, however, that the attributable fraction method relies on survey data on Swedish drinking patterns and also official Swedish data on the prevalence of potentially alcohol attributable diseases and injuries. A significant further limitation was that we only formally estimated confidence intervals around our estimates of alcohol consumption change and not around our estimates of how this translated into changes into alcohol attributable morbidity and mortality. Confidence intervals around estimates from the ARIMA models are shown in Table 4 but were not used to calculate the confidence intervals around our final estimates of changes in harm. Also, the time of writing, InterMAHP (Sherk et al., 2018) does not include a function to calculate confidence intervals. This will be addressed in a future version. It is likely, therefore, that the reported confidence intervals here are conservative.

We were unable to find an empirical basis upon which to estimate the effects on population consumption of the established tendency for private liquor stores to be less strict in their checking of customer age-IDs and level of intoxication than is the case in government-owned stores [20]. Neither did we take account of increased frequency of exposure for consumers to alcohol marketing and purchasing opportunities when visiting grocery stores for other items. We were also not able to include some 100% alcohol caused deaths e.g. cases of alcoholic gastritis from the general category of gastritis. These issues may have caused the estimates to underestimate the true impact of the changes.

While we acknowledge these various sources of possibly upward or downward bias in our estimation methods, a comparison with levels of consumption in other European countries shows that Sweden currently tends to have lower consumption than countries where alcohol distribution is fully privatised. In particular, we note per capita consumption levels of between 11 and 12 l per person aged 15+ in neighbouring Denmark and Germany which suggests our estimates are quite plausible. Furthermore, our estimates are based on the best-available evidence, draw on robust analytical methods and were subjected to examination of uncertainty.

### Implications for Swedish alcohol policy

Our results suggest abolishing Systembolaget would lead to significant increases in alcohol consumption and in the health and (some) social problems caused by alcohol. This is the case in both of the scenarios we examined which cover more or less restrictive visions of privatisation. This is because privatisation typically leads to a reduction in the minimum price charged for alcohol, an increase in the number of outlets selling alcohol, an increase the trading hours of those outlets and increased promotion and marketing of alcohol.

In theory it is possible to implement policies which would mitigate these effects and thereby prevent an increase in alcohol-related harms following privatisation. In practice, this has proved difficult to achieve in other countries with privatised alcohol markets as the number of commercial actors within the policy-making process tends to be both more numerous and effective in their lobbying efforts than in monopoly states. This has tended to stifle efforts to implement effective alcohol control policies and, conversely, has facilitated deregulatory measures that increase the potential for harmful public health consequences. The UK’s experience with minimum unit pricing for alcohol illustrates this point. Industry-led legal battles, for example, delayed the Scottish Parliament’s 2012 decision to introduce minimum unit pricing by six years [56]. By contrast, government alcohol monopoly jurisdictions can both introduce and modify all liquor prices at will by regulation with minimal delays (e.g. [37]). Given this and other experiences, it should not be assumed that a privatised market can be or will be straightforwardly and effectively regulated.

A government monopoly, especially one like Systembolaget with an explicit public health mandate, may be an ideal vehicle for enabling evidence-based alcohol policies to be implemented in the public interest. Nonetheless, we suggest Systembolaget could be used to generate further improved outcomes by having its policies strengthened in some areas e.g. by introducing an explicit minimum price per standard drink (12 g ethanol) for all alcoholic beverages indexed to the cost of living. It is possible to have both relatively high average prices for alcohol alongside quite low minimum prices, which is currently the case in Sweden. Thus setting minimum prices per standard drink and indexing these to the cost of living would further improve public health outcomes. In addition, any policy that increases competition in the alcohol market in Sweden is likely to have an adverse effect on public health and safety by driving down minimum prices even further and by increasing access, especially to under-aged drinkers. If Swedes wish to have an alcohol monopoly as an efficient tool to reduce harms, it is also important to not erode it through seemingly minor exceptions e.g. allowing alcohol sales via the Internet or permitting the sale of alcohol at farms, something currently being proposed.

### Recommendations for future research

Finally, we suggest that the research basis upon which estimates of the public health and safety impacts of alcohol policy changes are made needs to be strengthened. We highlight in particular the need for improved estimates of the risk relationships between alcohol use and disease based on longitudinal studies that control for different sources of lifetime selection bias e.g. bias caused by comparing risks for current versus former drinkers [57]. Similarly, improved methods are needed to estimate more precisely the relationships between drinking patterns in a population and the rate of acute alcohol-related harms.

In addition, a larger pool of well-controlled studies of the public health and safety impacts of abrupt changes in alcohol policies is needed, including studies which examine the interplay between multiple policy changes. An improved evidence base in each of these areas will support more precise estimates of the potential impact of hypothetical policy changes in a given jurisdiction.

## Conclusions

New understandings about how the distribution of alcohol consumption changes in a population as total consumption changes can be used also to help estimate changes in alcohol attributable harm under different policy scenarios. In depth studies of the relationship between per capita alcohol consumption and related harms in a country over many years can also be used for this same purpose. In the case of modelling estimated changes in alcohol related mortality as a result of privatising the Swedish government alcohol monopoly, the two methods produced broadly similar estimates of increased alcohol attributable harms. Confidence in this conclusion is supported by the degree of convergence in the estimates of increased harm from two quite different theoretical and methodological approaches. Although we have modelled the uncertainties due to random variation and presented these in our range of estimates, we have not modelled the impact of changing the assumptions upon which the model is based, and these may have a larger impact on the outcomes predicted by the model than the impacts of random variation.

While both privatisation scenarios considered resulted in substantial increases in alcohol consumption, attributable crime, hospitalisation and death, the largest increase was estimated for the sale of alcohol in grocery stores. We also conclude that improved health and safety outcomes could be achieved were Systembolaget to introduce still stronger policies, especially in the area of alcohol pricing. With increasing trends towards privatisation of alcohol control and distributions systems in North America, these estimates may also be a cautionary tale for policy makers in other full or partial alcohol monopoly jurisdictions. Increased government control over the distribution and sale of alcohol is also an option for countries with fully privatised systems to consider as an effective means of reducing alcohol-related harms.

## Appendix

**Table 6:** Three digit ICD-10 codes corresponding to alcohol attributable conditions used in GBD WHO method (Method A)

Major Category	Condition	ICD-10 code
Infectious diseases	Tuberculosis	A15 to A19
	HIV	B20 to B24
	Lower respiratory tract infections	J09 to J22
Cancer	Oropharyngeal cancer	C00 to C14
	Oesophageal cancer	C15
	Colorectal cancer	C18 to C21
	Liver cancer	C22
	Pancreatic cancer	C25
	Laryngeal cancer	C32
	Breast cancer	C50
Type 2 diabetes	Type 2 Diabetes mellitus	E11, E14
Mental health conditions	Mental and behavioural disorders due to alcohol	F10
	Epilepsy	G40 to G41
Cardiovascular conditions	Hypertensive disease / hypertension	I10 to I15
	Ischaemic heart disease	I20 to I25
	Cardiac arrhythmia	I47 to I49
	Heart failure and complications of heart disease	I50 to I52
	Ischaemic stroke	I63, I65 to I67
	Haemorrhagic stroke	CI60 to I62
Digestive conditions	Cirrhosis of the liver	K70, K74
	Acute pancreatitis	K85
Injuries*	Unintentional injuries	Begins with V or W, X00 to X59, Y40 to Y86, Y88, Y89
	Intentional self-harm	X60 to X84
	Assault/homicide	X85 to Y09

*ICD10 codes for injury hospital stays appear in an additional diagnosis category called “external cause of injury.” To be included, the primary diagnosis must have an ICD10 code in S00 to S99, T00 to T77, T79

**Table 7:** Causes of death and police-reported offences used in Method B

	ICD9	ICD10
Deaths		
Alcoholic liver disease	571.0–571.3	K70-K70.4, K70.9
Suicide	E950-E959	X60-X84, (except X65) Y87.0
Injuries. Composite measure comprising:		
*Drowning injuries*	E910	W65-W74
*Fall injuries*	E880-E888, E848	W00-W19
*Fire injuries*	E890-E899	X00-X09
*Motor-vehicle traffic crashes*	E810-E819	V02-V04, V12-V14, V20-V79, V89.2
*Undetermined*	E980–E989	Y10–Y34,Y87.2,Y89.9
Police-reported offences		
Assaults		
Drink driving		

**Table 8:** Estimated number of alcohol-attributable deaths in Sweden under Scenarios 1 and 2, by disease category, age group and gender

		Male			Female			Total		
		AAD	Scen1	Scen2	AAD	Scen1	Scen2	AAD	Scen1	Scen2
Cancers	15–34	2	3	3	2	3	3	4	6	6
	35–64	143	174	192	78	96	106	221	270	298
	65+	341	402	438	146	173	188	487	575	626
	Subtotal	486	579	633	226	271	297	712	850	930
Mental health conditions	15–34	2	3	3	1	1	1	3	4	4
	35–64	73	87	93	26	31	33	99	118	126
	65+	115	138	148	27	33	35	142	171	183
	Subtotal	190	228	244	54	65	69	244	293	313
Cardiovascular conditions	15–34	4	5	6	2	3	4	6	8	10
	35–64	72	141	183	16	35	48	88	176	231
	65+	−211	−81	11	− 335	−250	− 188	− 546	− 331	− 177
	Subtotal	− 135	65	200	− 317	− 212	− 137	−452	−147	63
Digestive conditions	15–34	1	2	2	0	0	0	1	2	2
	35–64	163	241	291	64	77	86	227	318	377
	65+	100	132	154	66	76	83	166	208	237
	Subtotal	265	374	446	129	154	168	394	528	614
Injuries	15–34	172	197	210	32	37	40	204	234	250
	35–64	218	256	277	44	52	57	262	308	334
	65+	145	177	194	41	51	56	186	228	250
	Subtotal	535	630	681	116	140	153	651	770	834
Infectious diseases	15–34	1	1	1	0	0	0	1	1	1
	35–64	6	7	8	2	3	3	8	10	11
	65+	48	59	65	23	28	31	71	87	96
	Subtotal	55	67	74	25	31	34	80	98	108
Type 2 diabetes	15–34	0	0	0	−1	−1	−1	−1	−1	−1
	35–64	2	3	3	−9	−10	−10	−7	−7	−7
	65+	10	12	13	− 136	−144	− 147	−126	− 132	− 134
	Subtotal	13	15	16	− 146	− 154	− 158	− 133	− 139	− 142
Total for all conditions	15–34	182	211	225	36	44	48	218	255	273
	35–64	676	909	1048	219	284	323	895	1193	1371
	65+	549	839	1022	− 168	−34	56	381	805	1078
	Subtotal	1408	1958	2294	87	294	427	1495	2252	2721

NB: Rows and columns may not add exactly due to rounding. AAD = alcohol-attributable deaths. Scen1 = predicted increase in deaths in Scenario 1. Scen2 = predicted increase in deaths in Scenario 2

**Table 9:** Estimated number of alcohol-attributable hospital stays in Sweden under Scenarios 1 and 2, by disease category, age group and gender

		Male			Female			Total		
		AAH	Scen1	Scen2	AAH	Scen1	Scen2	AAH	Scen1	Scen2
Cancers	15 to 34	29	35	36	30	38	43	59	73	79
	35 to 64	800	952	999	591	734	819	1391	1686	1818
	65+	1079	1234	1283	540	646	707	1619	1880	1990
	Subtotal	1908	2221	2318	1160	1417	1569	3068	3638	3887
Mental health conditions	15 to 34	2793	3216	3321	1920	2313	2473	4713	5529	5794
	35 to 64	14,042	16,287	16,841	4792	5765	6155	18,834	22,052	22,996
	65+	3685	4287	4438	939	1129	1208	4624	5416	5646
	Subtotal	20,520	23,790	24,600	7652	9207	9837	28,172	32,997	34,437
Cardio-vascular conditions	15 to 34	139	164	172	33	53	65	172	217	237
	35 to 64	340	728	845	− 891	− 682	− 550	− 551	46	295
	65+	− 2296	− 1825	− 1676	− 5258	− 5072	− 4932	− 7554	− 6897	− 6608
	Subtotal	− 1818	− 933	− 659	− 6117	− 5701	− 5417	− 7935	− 6634	− 6076
Digestive conditions	15 to 34	140	170	179	68	119	158	208	289	337
	35 to 64	904	1095	1155	267	362	425	1171	1457	1580
	65+	452	521	543	141	164	181	593	685	724
	Subtotal	1496	1785	1877	476	646	763	1972	2431	2640
Injuries	15 to 34	2765	3076	3175	1312	1542	1665	4077	4618	4840
	35 to 64	2822	3210	3324	1008	1214	1329	3830	4424	4653
	65+	1651	1925	2008	1008	1230	1358	2659	3155	3366
	Subtotal	7237	8210	8508	3328	3986	4352	10,565	12,196	12,860
Infectious diseases	15 to 34	164	195	204	108	136	153	272	331	357
	35 to 64	430	507	531	209	255	282	639	762	813
	65+	922	1075	1122	416	502	551	1338	1577	1673
	Subtotal	1516	1777	1857	733	894	987	2249	2671	2844
Type 2 diabetes	15 to 34	2	2	2	−11	−12	−12	−9	−10	−10
	35 to 64	26	30	31	−123	− 128	− 130	−97	−98	−99
	65+	30	33	34	− 297	−313	−321	− 267	− 280	−287
	Subtotal	58	65	68	−431	− 453	− 463	−373	−388	− 395
Total for all conditions	15 to 34	6031	6857	7089	3459	4190	4545	9490	11,047	11,634
	35 to 64	19,364	22,807	23,727	5852	7520	8331	25,216	30,327	32,058
	65+	5522	7251	7753	− 2511	− 1714	− 1248	3011	5537	6505
	Subtotal	30,917	36,915	38,569	6801	9997	11,628	37,718	46,912	50,197

NB: rows and columns may not add exactly due to rounding. AAH = alcohol-attributable hospital stays. Scen1 = predicted increase in hospital stays in Scenario 1. Scen2 = predicted increase in hospital stays in Scenario 2

**Table 10:** Estimated number of alcohol-attributable deaths in Sweden under Scenarios 1 and 2, by age group and gender for sub-groups of conditions with or without some assumed protection from alcohol

		Male			Female			Total		
		AAD	Scen1	Scen2	AAD	Scen1	Scen2	AAD	Scen1	Scen2
Sub-total for conditions with no protection	15 to 34	178	206	219	35	41	44	213	247	263
	35 to 64	603	765	861	214	259	285	817	1024	1146
	65+	749	908	999	303	361	393	1052	1269	1392
	**Subtotal**	**1531**	**1878**	**2078**	**550**	**661**	**721**	**2081**	**2539**	**2799**
Sub-total for conditions with some protection	15 to 34	4	5	6	1	2	3	5	7	9
	35 to 64	74	144	186	7	25	38	81	169	224
	65+	−201	−69	24	− 471	− 394	−335	− 672	−463	− 311
	**Subtotal**	**−122**	**80**	**216**	**−463**	**−366**	**− 295**	**− 585**	**− 286**	**−79**
Net total deaths for all conditions	15 to 34	182	211	225	36	44	48	218	255	273
	35 to 64	676	909	1048	219	284	323	895	1193	1371
	65+	549	839	1022	−168	−34	56	381	805	1078
	**Subtotal**	**1408**	**1958**	**2294**	**87**	**294**	**427**	**1495**	**2252**	**2721**

NB: rows and columns may not add exactly due to rounding. AAD = alcohol-attributable deaths. Scen1 = predicted increase in hospital stays in Scenario 1. Scen2 = predicted increase in hospital stays in Scenario 2

**Table 11:** Estimated number of alcohol-attributable hospital stays in Sweden under Scenarios 1 and 2, by age group and gender for sub-groups of conditions with or without some assumed protection from alcohol

		Male			Female			Total		
		AAH	Scen1	Scen2	AAH	Scen1	Scen2	AAH	Scen1	Scen2
Sub-total for conditions with no protection	15 to 34	5891	6692	6915	3438	4148	4492	9329	10,840	11,407
	35 to 64	18,998	22,051	22,850	6867	8330	9010	25,865	30,381	31,860
	65+	7789	9042	9394	3044	3671	4005	10,833	12,713	13,399
	**Subtotal**	**32,677**	**37,783**	**39,160**	**13,349**	**16,150**	**17,508**	**46,026**	**53,933**	**56,668**
Sub-total for conditions with some protection	15 to 34	141	166	174	22	41	53	163	207	227
	35 to 64	366	758	876	− 1014	−810	−680	− 648	−52	196
	65+	− 2266	− 1792	− 1642	− 5555	− 5385	− 5253	− 7821	− 7177	− 6895
	**Subtotal**	**− 1760**	**− 868**	**− 591**	**− 6548**	**− 6154**	**− 5880**	**− 8308**	**− 7022**	**− 6471**
Net total stays for all conditions	15 to 34	6031	6857	7089	3459	4190	4545	9490	11,047	11,634
	35 to 64	19,364	22,807	23,727	5852	7520	8331	25,216	30,327	32,058
	65+	5522	7251	7753	−2511	−1714	−1248	3011	5537	6505
	**Subtotal**	**30,917**	**36,915**	**38,569**	**6801**	**9997**	**11,628**	**37,718**	**46,912**	**50,197**

NB: rows and columns may not add exactly due to rounding. AAH = alcohol-attributable hospital stays. Scen1 = predicted increase in hospital stays in Scenario 1. Scen2 = predicted increase in hospital stays in Scenario 2

## Abbreviations

ECM: Error-correction modelling
GBD: Global Burden of Disease
InterMAHP: International Model of Alcohol Harms and Policies
SARIMA: Seasonal Autoregressive Integrated Moving Average Model
